# Associations of Liver Fluke Infection and Cholangiocarcinoma: A Scoping Review

**DOI:** 10.7759/cureus.46400

**Published:** 2023-10-03

**Authors:** Ankitha Sivanand, Durva Talati, Yash Kalariya, Priyansh Patel, Siddharth Kamal Gandhi

**Affiliations:** 1 Department of Internal Medicine, Civil Hospital Ahmedabad, Ahmedabad, IND; 2 Department of Internal Medicine, Medical College Baroda, Vadodara, IND; 3 Department of Internal Medicine, M.P. Shah Government Medical College, Jamnagar, IND

**Keywords:** associations, opisthorchis viverrinni, clonorchis sinensis, cholangiocarcinoma, liver fluke infection

## Abstract

Cholangiocarcinoma (CCa) is a highly lethal malignancy of biliary tract epithelial cells. Liver fluke infection is one of the well-known causes of CCa in endemic areas of Southeast Asian and Western Pacific regions. Multistep processes underlie carcinogenesis induced by chronic infection with the fish-borne liver fluke. Mechanical injury from fluke feeding and migrating in the bile duct causes damage to the bile duct epithelial cells. The excretory or secretory product of a parasite called OvGRN-1 is internalized by human cholangiocytes and induces changes in gene and protein expression associated with wound healing and cancer pathways. Inflammatory cytokines and their gene polymorphisms may also be linked to biliary pathologies. High plasma levels of interleukin 6 (IL-6) increase the risk of developing advanced periductal fibrosis (APF) and CCa by promoting CCa cell line proliferation. Anti-helminthic drugs can help decrease the risk of CCa caused by flukes. Surgical resection of the tumor and liver transplantation might be helpful too. Chemotherapy is considered for patients with advanced CCa when they cannot undergo surgery or when other treatment options fail to show improvement. Improvements in hygiene, health education, screening for fluke infection, and anti-helminthic therapy can help prevent liver fluke infection and thus the occurrence of CCa.

## Introduction and background

Cholangiocarcinoma (CCa) accounts for about 10% to 20% of primary liver malignancies worldwide [1]. It is the malignancy of epithelial cells anywhere along the biliary tract or even in the liver parenchyma, and it is highly lethal [2]. Infection caused by liver fluke is associated with CCa in endemic regions, mainly Southeast Asian countries [2]. In other regions of the world, there is usually no identifiable cause of CCa, but inflammation due to cholelithiasis, primary sclerosing cholangitis (PSC), and choledocholithiasis has been identified as the potential cause of CCa in some cases [2,3]. A case-control study conducted in China also showed that infection with the hepatitis B virus can be a possible risk factor for CCa development [4]. Patients are usually diagnosed with CCa in the advanced stages of the tumor, which makes it a difficult condition to cure, thus rendering the prognosis very poor [5]. CCa is resistant to radiation therapy and conventional chemotherapy [6]. Hence, the treatment options for it are narrowed down to surgical resection of the tumor [6]. But most of the time, metastasis to distant organs, lymph nodes, and blood vessels is often present during the diagnosis of CCa, which makes it a very aggressive tumor to resect [6]. The survival time is less than 12 months after diagnosis in people with an unresectable tumor [6].

More than 750 million people globally are at risk of being infected with liver fluke, including mainly people belonging to Southeast Asia and Western Pacific regions [7]. The three well-known flukes known to cause infection in humans are *Opisthorchis viverrini*, *O. felineus*, and *Clonorchis sinensis*, and their mode of infection is through ingestion of freshwater raw fish that is infested by the fluke [8]. Vietnam, Laos, Thailand, and Cambodia are endemic to the *O. viverrini* infection, whereas rural areas of China and Korea are endemic to the *C. sinensis*-associated infection [9]. The highest incidence of CCa and the highest prevalence of *O. viverrini* infection in the world are recorded in the Khon Kaen province of Northeast Thailand, suggesting a very strong correlation between CCa development and *O. viverrini* infection [10-13]. The carcinogenesis pathway via liver fluke infection consists of immunological and cellular reactions to the antigens and the excretory or secretory products of liver fluke, damage to the epithelium of the bile duct, changes induced by liver fluke in the bile duct, and the effects of repeated treatment of liver fluke infections [1].

Methodology

We chose PubMed, Medline, and PubMed Central databases for our data collection. We found 174 articles using regular keyword searches using the medical subject headings (MeSH) search criteria. The filters applied were "English language," "free full text," "animal species," and "human species." All the articles were screened through their titles and abstracts, and a few articles were shortlisted. The shortlisted articles underwent another evaluation, wherein the entire article was read thoroughly and only relevant articles were chosen. Finally, 21 articles were chosen for our article after the screening process.

## Review

Life cycle

**Figure 1:**
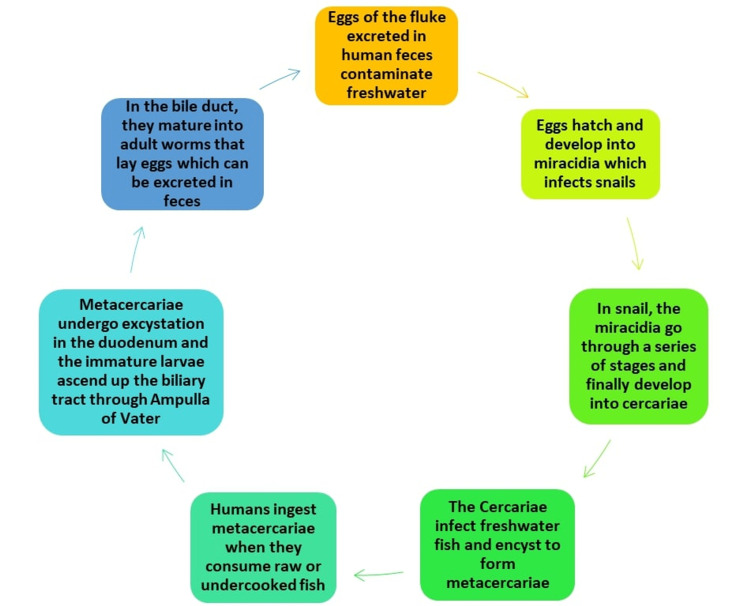
Life cycle of liver fluke Image credits: Ankitha Sivanand and Priyansh Patel

The life cycle of the liver flukes *O. viverrini* and *C. sinensis* starts when humans, who are the definitive hosts, pass the eggs of the fluke in their feces into the environment [1]. In freshwater, these eggs hatch and develop into miracidia, which infect snails (Parafossarulus or Bithynia species), which are considered the first intermediate hosts [1]. After infecting the snail, the miracidia first transform into a sporocyst, then into rediae, and subsequently into cercariae [1]. The cercariae then go on to infect the second intermediate host, which is the freshwater fish (Puntius species, Hampala, or Cyclocheilichthys species) [1]. They then undergo encystation in the muscles or under the scales of the fish and transform into metacercariae, which is considered the infectious stage [1,7,10]. Thus, humans get infected by consuming raw or undercooked fish containing the metacercarial stage of the parasite [1,10,13,14]. The encysted metacercaria is then digested by gastric and intestinal juices and undergoes excystation in the duodenum. The juvenile worm ascends through the ampulla of the Vater into the bile duct, where it matures into adult worms and deposits eggs, which are then passed on in the feces, thus repeating the cycle [1,2,7]. The amount of time that the parasite lives in the human body can go up to 25 years [1] (Figure 1).

Pathogenesis

Chronic liver fluke infection is considered one of the major risk factors for the development of CCa [5]. The continuous and prolonged inflammation caused by fluke infection can expose the bile duct epithelium to various carcinogenic substances and chemicals, which can lead to epigenetic and genetic damage in these cells. The accumulation of these abnormally damaged cells can lead to the development of CCa [5]. The main pathologic mechanisms of tumor formation are mechanical damage, excretory or secretory products of flukes, and immunological factors [1,9].

Mechanical Damage

The feeding and migrating activity of flukes can contribute to mechanical injury of the bile duct epithelium [9]. The ventral and oral suckers of the liver fluke hook onto the bile duct epithelium and can cause tissue damage, which can later ulcerate as the parasite matures [9,10]. The eggs of the fluke can get trapped in the ulcer, which can lead to the formation of granulomatous inflammation of the periductal tissue [1,9]. *C. sinensis* is relatively larger in size compared to the human bile duct, and hence it has the potential to cause partial obstruction of the bile duct, which can result in increased biliary pressure and stasis of the bile [1]. *O. viverrini* does not have the same effect as it is relatively smaller than *C. sinensis*. Eventually, deoxyribonucleic acid (DNA) damage and the development of CCa ensue due to repeated cycles of ulceration, granulomatous inflammation, and healing [1].

Excretory or Secretory Products

The liver fluke secretes a few metabolic products from its tegument or excretes products from its excretory openings into the bile, and some of these products can be highly immunogenic, which can play a role in the pathophysiology of CCa [9,10]. Apart from triggering immune reactions, the excretory or secretory products of the parasite can interact with the biliary epithelium and cause proliferation, differentiation, and several transcriptional changes in biliary cells [1,9]. In a study conducted in murine fibroblast cell lines that were cultured along with *O. viverrini*, the *O. viverrini* excretory or secretory products (OvESP) caused upregulation of gene expression in many pathways, especially involving transforming growth factor (TGF-β) and epidermal growth factor (EGF) [1,9]. A study investigating proteins in OvESP showed that it was made up of a complex mix of proteins like granulin, thioredoxin, and cystatin [1]. *O. viverrini* granulin (OvGRN-1), one of the major growth factors of OvESP, was considered homologous to human granulin and promoted wound healing at the site of the feeding of the parasite, which diminished the bile duct injury that the parasite initially caused [1,10]. According to an in vitro study, there was a proliferation of murine fibroblasts (NIH-3T3) and a human CCa cell line (KKU-100) stimulated by OvGRN-1 [1]. Antibodies directed at recombinant OvGRN-1 and ribonucleic acid (RNA) interference-induced suppression of OvGRN-1 expression reduced the proliferating capacity of OvESP on murine fibroblasts and host cells, indicating the vital role of OvGRN-1 in inducing carcinogenesis and in the establishment of a tumorigenic environment [1,15]. Another constituent of OvESP called thioredoxin (OvTrx-1), which has the capability of inducing inflammation, is an oxidoreductase enzyme that helps in combating the oxidative damage that occurs via the human immune response [1]. According to an immortalized human cholangiocyte cell line (H69) study, OvTrx-1 was found to inhibit apoptosis of the epithelial cells of the bile duct, induced by oxidative stress, which may play a role in the pathogenesis of CCa [1].

In a study conducted to gather more data on the mechanism by which cells internalize OvESP, three types of cells were incubated with OvESP: normal human cholangiocytes (H69), human CCa cells (KKU-100, KKU-M156), and human colon cancer cells (Caco-2). OvESPs were preferentially internalized by liver cells, specifically H69 cells [16]. Fluorescence techniques were used to detect any movement of the ESPs into the cholangiocyte organelles, and it was concluded that most of the ESPs stayed in the cytoplasm and were not getting transported to the organelles of the cell (as most fluorescence was detected in the cytoplasm). Analysis was also done on the different inhibitors of endocytosis to further strengthen the theory of OvESP internalization [16]. There was reduced endocytosis of OvESPs, especially in H69 cells, when the cells were pretreated with clathrin inhibitors. Clathrin and caveolae inhibitors (inhibitors of classical endocytosis) were able to block this proliferation, hence proving the mechanism of internalization and action of OvESPs [16]. It was also noted that OvESPs only induced liver cell proliferation (CCa cell lines and H69 cells) and not intestinal cell (Caco-2) proliferation [16]. A study conducted on *C. sinensis* demonstrated the upregulation of various genes involved in carcinogenesis by *C. sinensis* ESP (CsESPs) and the downregulation of various genes that induce apoptosis in the human CCa cell line (HuCCT1) [1]. A HuCCT1 cell line study found the expression of cyclooxygenase (COX-2) being induced by CsESPs and an increase in the proliferation of CCa cells in the presence of CsESPs. CCa cells that were treated beforehand with CsESPs were found to exhibit resistance to an anti-cancer and anti-inflammatory drug called Parthenolide, which functions to induce apoptosis of CCa cells [1]. CsESPs caused the downregulation of several microRNAs with tumor suppressor functions and upregulation of various cell proliferation-regulating microRNAs in not only the human CCa cell line (HuCCT1) but also the cholangiocyte cell line (H69), playing a role in the carcinogenesis process [1]. Another study demonstrated that CsESPs can cause histone modifications that may alter transcriptional modification of carcinogenic target genes like mcm7, which underlies the mechanism of hyperplasia in normal biliary cells, leading to the formation of adenomatous cells, which may further go on to transform into carcinoma cells [17]. Hence, it can be concluded that CsESPs cause changes in the proteome, transcriptome, and microRNA expression in HuCCT1 cells [17].

Immunological Factors

Mechanical damage by the flukes added with the reaction of the human immune system to the parasite causes extensive damage to the biliary epithelium [1]. Immunohistochemical staining of liver sections from hamsters that were experimentally infected with *O. viverrini* shows intense inflammatory cell accumulation in the infected liver sections [18]. Neutrophils, eosinophils, and macrophages comprise the early inflammatory stage, followed by plasma cells, macrophages and lymphocytes, and mast cells, which comprise the later stages of inflammation [18]. A case-control study determined the presence of advanced periductal fibrosis (APF) in asymptomatic individuals infected with *O. viverrini* and measured the cytokine response in them. The level of pro-inflammatory cytokine IL-6 in *O. viverrini* antigens was eightfold higher in people with APF than in people without APF [1,18]. This implies that IL-6 is one of the most important inflammatory cytokines involved in the immunopathogenesis of fluke-associated CCa [1].

The OvESP stimulates an inflammatory cascade by upregulating the toll-like receptor (TLR-4) and activating nuclear factor-kB (NF-kB) and secreting IL-6 and IL-8 [1,10,19]. The activation of NF-kB stimulates inducible nitric oxide synthase (iNOS) and COX-2 [1]. The nitric oxide (NO) that is released normally destroys pathogens but is considered mutagenic as it results in the release of nitrosamines, which is considered one of the initiators of CCa due to its oxidative nature that causes DNA damage [9,18]. Nitrosamines are commonly present in fermented food, which happens to be the staple food of Northeast Thailand [18]. These exogenous nitrosamines can become carcinogenic if fermented foods, like fermented fish, are ingested in large amounts [18]. In a study conducted, it was found that the pro-inflammatory cytokines IL-1b, IL-6, interferon (IFN-γ), leukotriene, and tumor necrosis factor (TNF-α) were significantly increased in patients with CCa with an underlying liver fluke infection than in patients with fluke infection and no CCa [19]. In another study, it was found that the pro-inflammatory cytokine IL-17A and the anti-apoptotic cytokine IL-22 were upregulated in liver fluke infection, and even higher levels were found in CCa associated with an underlying fluke infection [20]. Another mouse model study showed that there was an upregulation of TLR-2 and TLR-4 in endothelial cells, biliary epithelial cells, and fibroblasts due to *C. sinensis* infection, increasing IL-4, IL-10, IFN-γ, and TNF-α [1]. These studies suggest the role of inflammatory responses in fluke-infected patients in the development of CCa [19]. In conclusion, severe inflammation, DNA damage, and advanced periductal fibrosis are among the few immunopathologic mechanisms that contribute to the development of CCa [1].

Diagnosis

Due to the high prevalence of liver fluke in endemic areas, a screening test for fluke ova in stool is recommended along with abdominal ultrasound or other radiological imaging [2,8]. The stool ova test is relatively inexpensive and easily available [1]. In patients with negative stool ova tests, the polymerase chain reaction (PCR) method can be used for the detection of fluke genetic material in stool [1]. *O. vivverini* antigens can be found in the serum and can be used as a diagnostic tool. OvESP can also be detected in the urine of patients with fluke infections [1]. Diffuse dilatation of the intrahepatic bile ducts on ultrasonography or computed tomography (CT scan), in the absence of any obstructive cause, is one of the characteristic features of a previous or healed liver fluke infection [8]. Further screening and diagnostic tests should be conducted on these people to identify the present infection and/or CCa development [8].

Carbohydrate antigen 19-9 (CA 19-9) is one of the primary biomarkers for CCa and can be used as a diagnostic tool [2,8]. Magnetic resonance imaging (MRI) can produce an enhanced visual of the primary mass, whereas a CT scan can provide vascular enhancement, which can help to conclude if the tumor can be resected or not [2].

Treatment and prevention

Since liver fluke infection is one of the main causes of CCa development, it is important to treat this condition. Praziquantel, an anthelmintic drug, is usually the drug of choice for the treatment of opisthorchiasis and clonorchiasis [1]. Tribendimidine, an amidantel derivative, is another new drug that is showing promising results. It is effective not only against liver flukes but also against some intestinal roundworms, and hence can be used when there is a co-infection with flukes and roundworms [1]. Praziquantel does decrease the risk of CCa from fluke infection, but reinfection is a common phenomenon. Thus, future vaccination strategies against the fluke might be an important way to tackle this condition [2,10]. Some patients with CCa are recommended surgical options like surgical resection of the tumor and liver transplantation [2].

Surgical Resection

Even though surgical resection can be a potentially curative option, most patients present with advanced or metastatic CCa, which can make the tumor unresectable [2]. Staging laparoscopy which can detect metastatic disease not apparent on imaging, is usually recommended in patients with high CA 19-9 levels, vascular invasion of the tumor, and peritoneal spread of the tumor. Lymph node status is an important prognostic factor in patients undergoing resection. Porta hepatis lymphadenectomy is usually performed along with resection of the tumor [2].

Liver Transplantation

A liver transplant can be an option for patients with a small tumor. Tumor recurrence can occur if there is any microvascular invasion or poor differentiation of the tumor. There is a high risk of recurrence when liver transplants are done in patients with large tumors [2].

Chemotherapy

Cisplatin and gemcitabine are used as first-line chemotherapeutic agents in patients with advanced, unresectable CCa [2]. Folinic acid, 5-fluorouracil, and oxaliplatin can be used as second-line drugs in people whose tumor has shown progression even when on first-line drugs. The regimen of gemcitabine and cisplatin combined with paclitaxel is currently undergoing investigation on their efficacy as first-line agents [2].

Future Targeted Interventions

S100P plays an important role in cancer. A study conducted on S100P showed that it was present at very high levels in the serum and bile of patients with CCa, and the S100P mRNA was highly expressed in CCa tumor tissues [11]. S100P was also reportedly causing resistance in cancer cells to chemotherapy. Therefore, blocking S100P might be a targeted intervention used to sensitize cancer cells to chemotherapy drugs and help in the treatment of CCa [11]. Cyclophilin A (CypA), a cytosolic protein that is the main intracellular target of the drug cyclosporin A, is found to be significantly upregulated in most of the tissues of people with CCa [6]. Suppression of CypA expression was found to decrease the growth of tumors in mouse models and the proliferation of CCa cells in vitro, and hence this can be used as a new therapeutic target for CCa [6]. A study conducted on glucose transporter 1 (GLUT 1) demonstrated a connection between the overexpression of GLUT 1 and the aggressiveness of CCa [21]. Silencing of GLUT 1 significantly reduced the tumor growth, expansion, migration, and invasion of CCa cell lines, thus highlighting the role of GLUT 1 in the carcinogenesis and tumor progression of fluke-associated CCa [21]. Targeting GLUT 1 can thus be a potential therapeutic intervention in combating CCa [21].

Strategies to control liver fluke infection

Routinely eating raw, undercooked, or fermented fish combined with poor sanitation facilities and practices contributes to the high incidence of fluke infection in endemic areas [1,2,9]. A combined strategy including proper cooking practices, health education, sanitation improvement, and mass screening for fluke infections, followed by immediate treatment with praziquantel if detected, can help tackle the problem at hand and halt the progression to CCa [1,2].

## Conclusions

Mechanical damage to bile duct epithelial cells, immunologic and cellular reactions to ESPs, and inflammatory cytokines play a key role in the pathogenesis of liver fluke-associated CCa. Liver fluke infection continues to expand, impacting millions of people, which shows that awareness of this infection is insufficient. Proper sanitation measures, health education, early and regular screening for the liver fluke, and treatment with anti-helminthic drugs might play a key role in preventing liver fluke infection, which can subsequently help bring down the incidence of CCa.
